# Causes of Irregularities
*Read before the Association of Dental Surgeons, Baltimore, October, 1894.


**Published:** 1894-11

**Authors:** 


### Editorial, Etc.

CAUSES OF IRREGULARITIES.*—Few dentists, probably, have any idea that the subject of "orthodontia," which is now receiving the attention of the best minds in the profession, was given little or no space in the books and writings of former centuries. How to satisfy the demand for that branch of dental practice which relates to the correction of irregularity of position of the human teeth is one of the problems that the modern dentist has been called upon to face; and the dental student, with the approval of the National Association of Dental Faculties, is now instructed in the evils associated with irregularity, the advisability of correction, the age at which correction may be begun, the movements to be produced, the rules governing extraction as related to orthodontia, the physiology of tooth movement, the materials and methods to be used in regulating, etc. Let me epitomize the theories respecting the causes of irregularities which form a rich field for research.

Hippocrates (500 B. C ) laid down the dictum: "The more teeth the longer life." Aristotle one hundred years later said: "The fewer teeth the shorter life."

In 1618 a writer (Hilkiah Crooke) on second dentition says: "The shearing (i. e., incisors) teeth, when they do break forth, do thrust the first shearers out before them and issue betwixt the first two, the second and the dog tooth that

---

*Read before the Association of Dental Surgeons, Baltimore, October, 1894.

is next unto them. But if the former teeth will not fall or be not pulled out, or if the latter issue before the first fall, then the latter make their way through new sockets and turn in the upper jaw outward, in the lower jaw inward, so that there seemed to arise a new row of teeth, and this indeed hath deceived many historians and anatomists also."

The writers of the 18th century speak of supernumerary teeth causing irregularity; say that all the teeth that exceed 32 in number may be regarded as supernumerary; and attribute irregularity of canines and incisors to extreme narrowness of jaws.

In the present century the writers emphasize thumb-sucking; retention of temporary teeth; sleeping with the mouth open, *civilized* man being the only animal named that keeps its mouth open during sleep; premature extraction of temporary teeth; excessive vaulting of palate due to arrest of development of the sphenoid bone or defective growth of the vomer bone; civilization (for disproportion and consequent irregularity in the teeth of men and animals in the wild state is seldom found) and the jaws of civilized men are more contracted than those of semi-barbarous races; intermarriage between persons of different nationalities, the children inheriting the contracted jaws of one parent and the large teeth of the other; nervous disturbances.

It would not be reasonable to class all deviations from the ideal type under irregularities; otherwise all peculiarities of race or nationality would have to be included. It is a recorded fact by Messrs. Coleman and Cartwright that the early races possessed large jaws and regular teeth: irregularity rarely if ever occurred, nor do irregularities prevail among members of the clannish tribes. Among the thousands of Chinese and Indians examined by Dr. Nichols in 12 years he failed to find a case of irregularity of the teeth. Where the population is made up of immigrants from every country deformities of the jaws and teeth are found, which increase with the growth of the country. America fitly expresses this condition, for in this country all the great races of the world, (European, African, Asiatic and American) are thrown together on a great scale; The result of a union of Indian and Negro with

Americans does not possess the distinguishing characteristics of either of the races, (but no longer showing the perfectly symmetrical jaws of the Indian, or the protruding maxilla of the Negro, but a smaller jaw,) is recognized as half-breeds, octoroons, etc.

"The laws of inheritance," says Kingsley, "confirmed by common observation, show how constant is the mingling in the offspring of the traits of character and the peculiar features of two diverse races brought together in marriage. This mixture, without blending or harmonizing is productive of deformity in character and physique. Thus, so far as the jaws and teeth are concerned, they may exist in each parent in perfect symmetry; in one parent the jaws and teeth are large; in the other parent both jaws and teeth are small; but each in its way is a normal development. If now the small jaw of one parent and the large teeth of the other appear in the offspring, deformity is sure to follow."

Enlargement of the jaw bones, due to herpertrophies, osteites, periostitis, disease of antrum and nasal fossæ, is an occasional cause of dental irregularities. The growth of the teeth does not proportionately increase and the consequent misproportion between the teeth and jaws necessarily produces a deformity. The forms of irregularities of the teeth that co-exist with crowded arches are not seen in enlarged jaws. Rachitis in children, whether due to syphilis or not, may be localized in some portion of the jaw, causing it to be unevenly developed. Measles and pneumonia are also said to be followed by dental irregularities and maxillary deformites.

Diathesis enters largely into the etiology of maxillary and dental deformities; and among the characteristics of strumous children noted by Fothergrill are ' teeth crowd, carious."

The superior maxilla shows indications of gradually diminishing in size. It is a fixed bone and the inaction from lack of exercise gradually affects and diminishes the volume of bone-tissue from one generation to another. The inferior jaw on the other hand, is constantly in motion. Exercise of the inferior maxilla, which has always existed, has developed the jaw, while the superior maxilla has dwarfed from non-exercise.

Asymmetry of the lateral halves of the maxillary bones exists in the present era of the human race. Each lateral half of the body develops independently of the other. The superior and inferior maxillary ones, growing independently of each other, may be subjected to peculiar characteristics of environment, so that the result of their development may be asymmetry of the jaws. Marked illustrations of asymmetry of the human body is seen in the blacksmith whose right arm is larger and will weigh heavier than the left, and in the peddler who in carrying a pack has the side most in use developed more than the other.

Dr. L. P. Haskell observed a lack of harmony in the lateral halves of the maxillary bones:

| | UPPER JAW. | | LOWER JAW. |
|---|---|---|---|
| No. Examined, | 298 | - | 154 |
| No. Normal, | 137 | - | 54 |
| Abnormal Right Side, | 73 | - | 12 |
| " Left " | 88 | - | 88 |

He says: "Every dentist of experience must have observed that greater length of teeth and gums is required upon the left side than upon the right."

Dr. E. S. Talbot, in the study of irregularities of the teeth, observed that although no two cases are exactly alike, there is a general similarity of shape and outline of alveolar process, and jaws owing to similar environments during the eruption of the teeth. Upon the hypothesis that the two halves of the superior maxilla are developed in proportion to the excess of food masticated on one side or the other, depending upon right and left handedness of the individual, he would conclude that if the left side of the jaw is larger, the person is left-handed. He says, "When we consider the complexity of the development of bone-tissues, first of the maxillary bone, then of the alveolar process, and lastly of the two sets of teeth, it is a wonder that harmony ever prevails."

In 1888 Dr. Louis Ottofy reported an examination of the mouths of 623 public school children living in the country —307 males and 306 females, with the following percentage of irregularities:

| AGE. | IRREGULAR. per cent. | REGULAR. per cent. |
|---|---|---|
| 5 | 0 | 100 |
| 6 | 9 | 91 |
| 7 | 27.5 | 72.5 |
| 8 | 43 | 57 |
| 9 | 14 | 86 |
| 10 | 31.5 | 68.5 |
| 11 | 32.5 | 67.5 |
| 12 | 25 | 75 |
| 13 | 20 | 80 |
| 14 | 35 | 65 |
| 15 | 28 | 72 |

It will be seen that the largest per cent. of irregularities is found at the age of 8 years. The cuspid teeth are appearing at this time, and at least one-half of the irregularities is due to local causes. At the age of 13 but 20 per cent. of the cases showed deformities; nature and judicious use of the forceps had corrected many of them. At the age of 15, 28 per cent. of the teeth were regular.

Dr. E. S. Talbot made an examination of the mouths of 1,000 children, 396 male, 604 female, over 12 years of age, mostly residents of Chicago who had been attended by the dentist regularly, and found 780 or 78 per cent. normal.

Dr. P. N. Moriarty conducted an examination of 223 persons from tne poorer classes seeking the services of the students of the Harvard Dental School, Boston, and found 60 per cent. normal. R. G.

